# Review of Microdevices for Hemozoin-Based Malaria Detection

**DOI:** 10.3390/bios12020110

**Published:** 2022-02-11

**Authors:** Vitória Baptista, Weng Kung Peng, Graça Minas, Maria Isabel Veiga, Susana O. Catarino

**Affiliations:** 1 Microelectromechanical Systems Research Unit (CMEMS-UMinho), School of Engineering, Campus de Azurém, University of Minho, 4800-058 Guimarães, Portugal; gminas@dei.uminho.pt (G.M.); scatarino@dei.uminho.pt (S.O.C.); 2 LABBELS-Associate Laboratory, Braga/Guimarães, 4806-909 Guimarães, Portugal; 3 Life and Health Sciences Research Institute (ICVS), School of Medicine, Campus de Gualtar, University of Minho, 4710-057 Braga, Portugal; mariaveiga@med.uminho.pt; 4 ICVS/3B’s-PT Government Associate Laboratory, Braga/Guimarães, 4806-909 Guimarães, Portugal; 5 Songshan Lake Materials Laboratory, Building A1, University Innovation Park, Dongguan 523808, China; pengwengkung@sslab.org.cn

**Keywords:** biosensor, diagnosis, hemozoin, lab-on-a-chip, malaria, microdevices

## Abstract

Despite being preventable and treatable, malaria still puts almost half of the world’s population at risk. Thus, prompt, accurate and sensitive malaria diagnosis is crucial for disease control and elimination. Optical microscopy and immuno-rapid tests are the standard malaria diagnostic methods in the field. However, these are time-consuming and fail to detect low-level parasitemia. Biosensors and lab-on-a-chip devices, as reported to different applications, usually offer high sensitivity, specificity, and ease of use at the point of care. Thus, these can be explored as an alternative for malaria diagnosis. Alongside malaria infection inside the human red blood cells, parasites consume host hemoglobin generating the hemozoin crystal as a by-product. Hemozoin is produced in all parasite species either in symptomatic and asymptomatic individuals. Furthermore, hemozoin crystals are produced as the parasites invade the red blood cells and their content relates to disease progression. Hemozoin is, therefore, a unique indicator of infection, being used as a malaria biomarker. Herein, the so-far developed biosensors and lab-on-a-chip devices aiming for malaria detection by targeting hemozoin as a biomarker are reviewed and discussed to fulfil all the medical demands for malaria management towards elimination.

## 1. Introduction

Malaria, which is transmitted by the bite of female Anopheles mosquitoes infected with *Plasmodium* parasites, is one of the most life-threatening infectious diseases worldwide, with a significant impact on human lives. Most malaria cases occur in tropical and sub-tropical developing countries, where poverty limits access to proper healthcare conditions and infrastructures [1]. The use of insecticide-treated nets and artemisinin-based combination therapies allowed noticeable progress to be made in the face of the burden of malaria [1]. Nevertheless, this progress levelled off in recent years. Malaria incidence and mortality decreased by 27% and 52%, respectively, from 2000 to 2015, and then around 2% and 16%, respectively, from 2015 to 2019 [1]. This decline most likely results from an increase in disease transmission due to mosquitoes and parasites growing resistance to insecticides and antimalarial drugs, respectively [1]. In fact, in 2020, the World Health Organization (WHO) still reported 241 million global malaria cases and around 627,000 deaths [2]. The COVID-19 pandemic, which undermined prevention, diagnosis, and treatment, is likely a major contributor to these devastating numbers, and there are calls for the approval of the first malaria vaccine, despite its modest efficacy [2,3].

Malaria may manifest as symptomatic or asymptomatic, despite parasites circulating in the patient bloodstream. Typically, symptomatic malaria includes fever, tiredness, digestive symptoms and shaking chills, which may progress, in severe cases, into a coma, seizures, cerebral malaria and even death [4]. Malaria symptoms are related to the intraerythrocytic stage of infection in the human host and, thus, this stage is the target for infection detection. Nevertheless, since these symptoms are common to other febrile illnesses, they are often neglected or misdiagnosed. Therefore, sensitive and specific malaria diagnostic techniques, able to strengthen the disease surveillance for better management and control, are a crucial step to achieve the United Nations Sustainable Development Goal 3 towards malaria elimination [5,6].

The ability to quantify and detect low-level infections (ideally less than 5 parasites/µL of blood) is of utmost importance, as it: (1) prevents the progression into severe disease and even death of the patient, (2) allows patients to be cleared after treatment and to identify emerging drug-resistant strains (identified by the inability of the drug to clear the parasites in three days) and (3) decreases inadequate treatment and prevents the emergence and spread of antimalarial drug resistance [7]. The conventional malaria diagnosis methods rely on optical microscopy of Giemsa-stained blood smears and rapid-diagnostic tests (RDTs) [1,8]. Optical microscopy allows parasite species and stage to be identified, and parasitemia quantification up to detection limits of 50–200 parasites/µL of blood. However, it is time-consuming, requires infrastructures that are not easily accessible in endemic areas and is highly microscopist-dependent. RDTs, which work on the principle of the detection of specific antigens produced by the malaria parasite, are portable and easy to use at the community level, reaching more patients. Nevertheless, these do not allow parasitemia quantification nor present a better limit of detection than microscopy, achieving only 100–200 parasites/µL of blood [9]. The most sensitive malaria detection (around 5 parasites/µL of blood) is achieved by nucleic acid-based detection methods. However, these are only performed in research settings since they require skilled personnel and high-rate equipment, difficult to reach in malaria-endemic regions [10]. Thus, the lack of on-field sensitive methods able to detect malaria and quantify parasitemia, coupled to rapid, easy to perform and low-cost detection, mean that there is a need for new diagnostic approaches for proper malaria control, the performance of which must be compared with the one of the gold-standard optical microscopy (below 50–100 parasites/μL of blood). In fact, the need for such a device has already led to the development and improvement of many novel technologies [11,12,13,14]. However, to date, none fulfil all the critical requirements regarding detection limits, sensitivity, specificity, portability, low cost, ease of use and, ideally, non-invasiveness [11,12,13,14]. This short literature review focuses on the most recent developments in biosensors and lab-on-a-chip devices for malaria detection, specifically on those using hemozoin as a biomarker. It also discusses the potential and limitations of these devices for diagnosis. To the best of the authors’ knowledge, there is no such review in literature discussing this topic, and, hence, it is expected that this review can bridge the gap.

## 2. Malaria Biomarkers

Biomarkers are biological characteristics that work as measurable indicators of normal and pathological processes and therapeutics response, providing information about the biological state of an organism [15]. Concerning malaria, there are plenty of indicators of infection. These include the enzymes *Plasmodium falciparum* histidine-rich protein II (*Pf*HRP-II), *Plasmodium* aldolase (*P*ALD), *Plasmodium* lactate dehydrogenase (*P*LDH), *Plasmodium falciparum* hypoxanthine-guanine phosphoribosyl transferase (*Pf*HGPRT) and *Plasmodium* glutamate dehydrogenase (*P*GDH) that are expressed upon infection [11,16,17]. In fact, most of the current RDTs for malaria detection, which correspond to a test strip with a nitrocellulose membrane comprising capture antibodies and antibodies against target antigens, target on *Pf*HRP-II, *P*ALD and *P*LDH detection [9]. *Pf*HRP-II is the most predominant target for *P. falciparum* infections; however, RDTs based on this fail to detect the emerging parasites that no longer express HRP-II [1]. Currently, no non-HRP-II-based RDTs are qualified to detect and distinguish between species [2]. Furthermore, some *P*ALD-based RDTS present poor sensitivity due to low expression of the enzyme by parasites [16]. As reviewed elsewhere, several biosensors, as well as lab-on-a-chip devices, are targeting these enzymes for malaria detection, as they are the best-known point-of-care RDTs [11,16]. Nevertheless, there is an urge for other technologies with improved sensitivities at the point of care. In this sense, new biosensors and lab-on-a-chip devices have been exploring other biomarkers for diagnosis, including hemozoin.

### Hemozoin: A Malaria Biomarker

**Figure 1:**
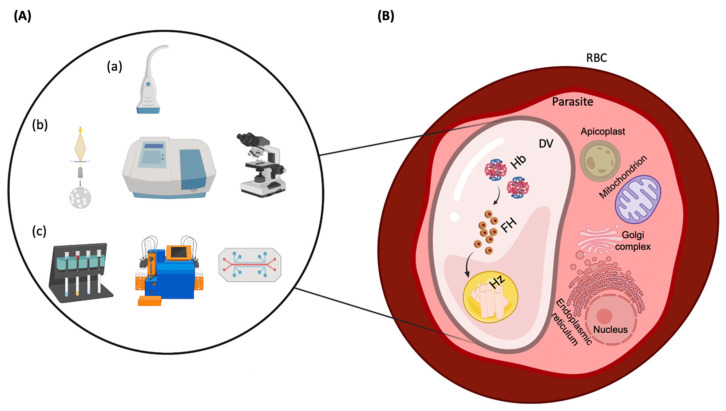
(**A**) Tools that allow the detection of (a) acoustic, (b) optical, (c) magnetic and electrochemical properties of hemozoin (Hz). (**B**) *Plasmodium*-infected red blood cell (RBC) with Hz formation occurring in the digestive vacuole (DV) of the parasite. As parasite invade RBCs, hemoglobin (Hb) is degraded, releasing free heme (FH) that is polymerized into Hz.

Hemozoin is an insoluble crystallite produced by malaria and malaria-non-related parasites [18,19,20,21]. During the intraerythrocytic stage of infection, parasites develop into different morphological structures passing from early-stage (rings) to late-stage (trophozoites and schizonts) structures and sometimes developing into sexual gametocytes that do not cause any clinical manifestation of the disease but are responsible for its transmission [22,23]. Additionally, along this intraerythrocytic stage, *Plasmodium* species rely on host hemoglobin (the main component of red blood cells (RBCs)) as a source of amino acids [24]. Notwithstanding, hemoglobin degradation releases free heme parts that cause oxidative stress to the parasite [24,25,26,27]. Thus, to evade heme toxicity and cell death, the parasite converts heme into an inert crystal, the hemozoin, in the digestive vacuole [26,27]. Considering Hole et al. and Pisciotta et al., 10^10^ parasites produce 3–4 µmol of hemozoin, which corresponds to around 0.4512 pg of hemozoin/parasite [28,29]. Thus, taking this into account, since hemozoin content increases with the progression of the disease, while being absent in healthy individuals, it is an important biomarker of infection [30]. Hemozoin, also known as the malaria pigment due to its brown pigmentation at standard light microscopy, consists of a polymer of five heme molecules, linked by bonds between the central ferric iron of one heme and the carboxylate group of another heme [31,32]. This structure resembles β-hematin, the synthetic hemozoin [32,33]. Although the hemozoin formation process is not entirely resolved, it is known that this crystal is optically birefringent and paramagnetic [34,35,36,37,38]. These unique features have become an attractive target for the development of new malaria detection methods [12,37,38,39,40,41,42,43,44,45,46]. Among others, these include micro and miniaturized devices that have been explored for hemozoin-based malaria detection aiming to achieve portable, low-cost and low-power consumption detection [21,42,45,47,48]. Within this review article, we explore these devices. In Section 3, we dissect the so-far developed biosensors (mainly electrochemical and optical biosensors) for hemozoin detection. As mentioned above, biosensors require a specific bio-recognition element, and thus, other label-free micro and miniaturized devices have been developed, including lab-on-a-chip devices. Lab-on-a-chip devices, described in Section 4, encompass a sensing technology (with or without the need of reagents or bio-recognition elements) coupled with microfluidics to separation, margination, and concentration of parasites, facilitating and increasing the sensitivity of subsequent sample analyzes. Figure 1 represents the pathway towards hemozoin formation, as well as techniques that allow its detection based on its unique optical, electrochemical, magnetic, and photoacoustic properties.

## 3. Biosensors for Hemozoin-Based Malaria Diagnosis

In a general view, biosensors are sensing devices that comprise a bio-recognition element and a transducer. The bio-recognition element (e.g., enzymes, antibodies, microorganisms, DNA) identifies and interacts with the analyte/target of interest, and alterations in its physicochemical properties (e.g., optical, piezoelectric, magnetic, electrochemical) are converted into a quantitative or semiquantitative measurable electrical signal by a transducer [49]. In recent years, due to their sensitivity, specificity and high-throughput screening, biosensors have had significant growth, aiming at a vast range of applications [50]. Specifically, in diagnosis, biosensors have been exploited for integration into point-of-care devices. The most commonly known biosensor is the glucometer, which measures the glucose levels in the blood and has greatly contributed to diabetes management [51]. Given its success, this technology has been widespread to other diseases, including malaria, which is the focus of this manuscript. The current major challenges of malaria screening are the need for point-of-care sensitive detection of low parasitemia. Ideally, such challenges and limitations can potentially be overcome with properly designed biosensors, that must fill the gap of high sensitivity and specificity, as well as being easily miniaturized for point-of-care diagnosis. Although there are several reports of biosensors for malaria diagnosis, these are mainly based on the use of enzymes as a target [11,16,17,52,53,54], i.e., RDTs. Nevertheless, as mentioned above, these have been threatened by parasite genetic evolution, and they do not detect low-parasitemia (100 parasites/µL of blood far from the ideal less than 5 parasites/µL of blood) or quantify infection. On the other hand, hemozoin: (1) is present in all parasite species, (2) is a crystal not being prone to genetic modifications, (3) is formed as parasites invade the patient’s RBCs and (4) its content relates to disease progression, i.e., increase in parasitemia, and hence disease severity. Thus, a specific bio-recognition element for hemozoin detection incorporated in biosensors can be a starting point to future point-of-care malaria diagnostic devices. Preferably, this device should not only detect hemozoin but also quantify it in an amount equivalent to 1 parasite/µL (0.4512 pg of hemozoin [28,29]). Herein, we explore hemozoin as a biomarker, summarize the recent advances in hemozoin-based biosensors (as listed in Table 1) and discuss their applicability.

### 3.1. Electrochemical Biosensors

Electrochemical biosensors detect an electrical signal when a biological analyte reacts with the surface of the sensor. The amplitude of the electrical signal correlates with the concentration of the analyte. These biosensors have the advantage of being highly sensitive and specific, low-cost, presenting a rapid response, allowing quantification and performing a simple assay [55]. However, these are thermo-sensible, with a narrow temperature range, and may present a short life span, with limited shelf time, due to the nature and stability of their biological analytes or number of interactions with their targets. The probability of nonspecific binding of the analytes continues to be one of the main limitations of these biosensors [56]. Due to their temperature sensitivity, these sensors typically include internal circuits for temperature compensation, which may increase their complexity.

Regarding the development of electrochemical biosensors aiming malaria detection, recently, Obisesan et al. [57] developed an electrochemical nanosensor for the detection of β-hematin, the synthetic hemozoin. The authors, chemically and by using a microwave, synthesized metal oxide nanoparticles of copper, iron and aluminium, and deposited them on a gold electrode by using the drop-dry method [57]. A metal oxide electrode disk was used as the working electrode, a platinum disk was used as the counter electrode and an Ag/AgCl, saturated KCl was used as the reference electrode, at a constant pH of 9.0 [57]. The electrochemical sensor was tested in human non-malaria-infected urine samples, human malaria-infected serum, as well as mice non-infected and infected serum, all mixed with β-hematin. Additionally, the behavior of each metal oxide-coated electrode was explored by a cyclic voltammetry experiment. The report shows that the gold-coated electrode with metal oxide nanoparticles, preferably with copper, presented improved electrode catalysis, high stability and sensitivity of high reduction current and lower energy towards malaria detection, thereby supporting the potential of these sensors for detection and quantification of malaria parasite in biological fluids [57]. In fact, the authors were able to detect and consistently quantify 3.50–4.8 mM and 0.65–1.35 mM of β-hematin in mice and human serum samples, respectively [57]. Considering the conversion of units as proposed by other literature reports (such as [28,29]), the amount detected by this system might not present a better detection limit than the standard methods (approximately 1.77 × 10^9^ – 3.68 × 10^9^ parasites/µL of blood for the blood samples quite far from the 50–200 parasites/µL and 100–200 parasites/µL of blood of microscopy and RDTs, respectively). Furthermore, stable electricity for the operation of devices (including microwave, sonicator, magnetic stirrer, centrifuge) is required for the preparation of nanoparticles and the electrode and processing of the sample before use. It is noteworthy that the authors show that the stability of the electrodes decreases due to an increase or decrease in current response at the electrode after 20 cycles. These, coupled with the expensive technology, might compromise the applicability of the method.

In an opinion article, Moutaouakil and colleagues dissect the properties of graphene, more specifically its electrical and optical properties, and propose the use of a graphene-based biosensor for malaria diagnosis [58]. The authors suggest that the flexible nature of graphene allows it to be integrated into PCBs to offer different commercial applications, including in RDTs and thick blood films [58]. Graphene is able to monitor the electronic transfer reactions of hemoglobin and, thus, may detect malaria infection [58]. This is because the conversion of hemoglobin in hemozoin causes oxidation of the iron from its ferrous state Fe^2+^ into its ferric state Fe^3+^, leading to electrons transfer [26,27]. In fact, it was demonstrated that RBCs are immobilized on a glassy carbon electrode surface and that, by cyclic voltammetry, a reduction peak was observed for hemoglobin [59]. However, despite the promising results, it remains unknown whether this can be applicable for hemozoin-based malaria detection.

### 3.2. Optical Biosensors

Optical biosensors are based on detecting changes in light upon the interaction between the bio-recognition element and the target and they include reflection, fluorescence, luminescence, optical fiber, photonic and surface plasma resonance (SPR) biosensors [49]. Among these, fluorescence and SPR biosensors are the most popular. Fluorescence sensors are based on the fluorescent light emission of fluorophore molecules at a specific wavelength, after the radiation absorption at a different energy level (lower wavelength). The fluorescence intensity is proportional to the concentration of the analyte and the sensor response can be measured either through intensity or decay-time sensing [60]. Although these sensors assure high sensitivity and specificity and are immune to light scattering, they are limited by the short life span of the fluorophores and their photostability, and they are susceptible to pH and oxygen interferences [60]. SPR biosensors measure alterations in the refractive index of the plasma resonance material, in the SPR angle and reflectance intensity, caused by the interaction between the bio-recognition element and the target [61]. These biosensors are highly sensitive, present high resolution, can be label-free and allow real-time measurements, as they are adequate for point-of-care applications. However, SPR sensors are motion-sensitive and depend on the development of light detectors with a high signal-to-noise ratio [62]. They require precise alignment between light and the sensing area, regarding both distance and angle, and their signal is dependent on the molecular size and concentration of the analyte. Additionally, when not fully automated and integrated, optical measurements need long calibration processes and are mainly limited by the time required for precise sample and setup preparation in order to avoid light interferences [60,63].

**Figure 2:**
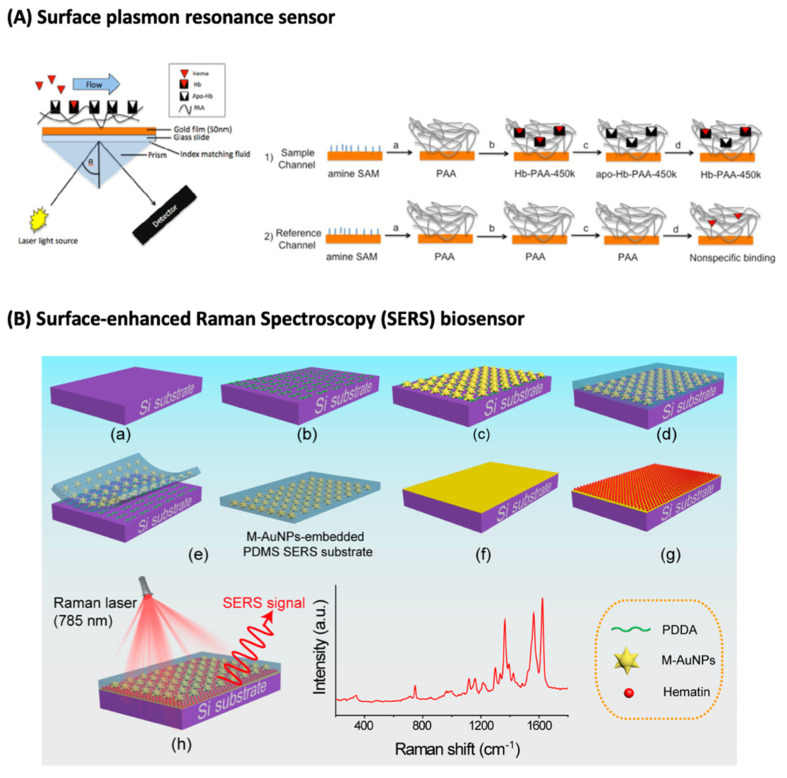
Example of sensors for malaria diagnosis based on hemozoin detection. (**A**) Schematic of a surface plasmon resonance sensor 1: (a) attachment of PAA to amine monolayer with EDC/NHS, (b) immobilization of Hemoglobin in PAA matrix, (c) removal of heme from hemoglobin and (d) injection of heme solution resulting in heme reconstitution 2: (a) attachment of PAA to amine SAM with EDC/NHS, (b) channel blocked off, (c) acid/acetone wash, and (d) injection of heme solution to determine nonspecific binding. (**B**) Schematic of the fabrication process of a SERS biosensor, where (a) Si substrate, (b) Si coated with PDDA, (c) M-AuNPs assembled on the PDDA-coated Si, (d) PDMS layer formed on the M-AuNP-assembled Si, (e) M-AuNP-embedded PDMS film, (f) Au film-coated Si, (g) hematin deposited on the Au film surface and (h) hematin-deposited Au film covered with M-AuNP-embedded PDMS SERS active substrate. Reprinted with permission: (**A**) [61] copyright © 2012 Elsevier B.V. All rights reserved, and (**B**) [75] copyright © 2021 American Chemical Society. Acronyms: Au, gold; EDC, 1-ethyl-3-(3-dimethylaminopropyl) carbodiimide; NHS, N-hydroxysuccinimide; NPs, Nanoparticles; PAA, Polyacrylic acid; PDDA, Poly(diallyl-dimethylammonium); PDMS, Polydimethylsiloxane; SAM, Self-assembled monolayer; SERS, Surface-enhanced Raman Spectroscopy; Si, Silicon.

Regarding optical biosensors for malaria detection, Briand and co-workers (Figure 2A) used hemoglobin-polyacrylic acid as a bio-recognition element for a gold-coated SPR-based sensor for rapid heme detection [61]. This biosensor acts by removing heme from hemoglobin, followed by heme-free hemoglobin exposure to samples containing heme that interact with the bio-recognition element. The authors were able to rapidly (less than 10 min) detect the presence of heme with a detection limit of 2 µM or 1.30 µg/mL with high selectivity, proving the method applicability [61]. As the authors measured heme concentration (an intervenient prior hemozoin formation), the values cannot be converted into parasite/µL. Furthermore, this device presents a good performance and good reusability, shown by the fact that it was used 12 times [61]. Additionally, for on-field applicability, other polymers in the SPR surface and an increase in the concentration of the biorecognition element might improve specificity as well as sensitivity [61].

Taking advantage of the different refractive index of infected and non-infected RBCs, Sharma et al. proposed a biosensor based on 2D photonic crystal, using a linear waveguide with a nanocavity to trap RBCs and detect shifts in the transmission peak at 1550 nm [64]. Bendib S. and Bendib C. also designed and simulated a 2D photonic crystal biosensor [65]. This simulated biosensor uses a sensitive increaser ring resonator based on GaAs rods of a rectangular lattice suspended in air background and was investigated by using plane wave expansion and finite difference time domain methods [65]. The authors relate the refractive index with the bandgap of infected (in ring, trophozoites and schizont stages) and non-infected RBCs to improve the sensitivity of the biosensor [65]. However, the authors do not specify the parasitemia of the infected samples used in the performed assays. Recently, Rashidnia and co-workers used the same principles and designed and simulated a 2D photonic crystal biosensor with a rectangular geometry of gold rods [66]. Ankita et al. proposed a simpler 1D PC photonic crystal biosensor with a defect layer also able to detect changes in the transmission peak, according to the concentration of hemoglobin in infected and non-infected blood samples [67]. While the fabrication of photonic crystals might be a challenge due to their precise structure and dimensions, there are several fabrication methods and materials that make them inexpensive.

Quite recently, Chaudhary et al. joined the technology of photonic crystals and SPR to develop a gold-immobilized photonic crystal fiber-based SPR biosensor for malaria detection [68]. This system measures changes in the RBCs refractive index, not specifically hemozoin [68]. Briefly, the sample is added into the photonic crystal fiber and an SPR shift in resonance wavelength, which is dependent on the refractive index, and is detected between healthy and infected RBCs at different stages [68].

Abshire et al. [69] developed a heme fluorescence-based biosensor that undergoes fluorescence quenching upon heme binding. To achieve this, the authors constructed a fluorescence resonance energy transfer (FRET)-based heme sensor, in which enhanced cyan and yellow fluorescent proteins act as the donor and acceptor, respectively, and PfHRP-II as the heme-binding domain [69]. By doing so, the authors were able to identify heme pools in *P. falciparum* by fluorescence microscopy and observe alterations in heme concentrations in the presence of the antimalarial drug chloroquine [69]. Nevertheless, this biosensor is not applicable on-field since the parasite must incorporate the FRET-based heme sensor for it to be used.

Surface-enhanced Raman spectroscopy (SERS), which enhances Raman signals, is another optical technique that is becoming popular due to its high sensitivity and specificity, as it is unaffected by temperature and humidity changes. It depends on the rotational and vibrational states within the molecules, as it is used to detect the specific absorption bands of different functional groups and quantify the corresponding molecules. However, this technique is susceptible to noise interferences due to its low signal-to-noise ratio [60]. Garret et al. [70] used gold-coated *Graphium weiskei* butterfly rings for the development of a SERS biosensor for malaria diagnosis by interaction with hemozoin. By doing so, the authors were able to detect a parasitemia of 0.0005% and 0.005% of lysed samples of early-stage *P. falciparum*-infected RBCs [70]. Assuming an RBC count of 5 × 10^6^/µL of blood [71], and of that 50% corresponds to RBCs, the parasitemia range obtained within this work is equivalent to the one of microscopy (50–500 parasites/µL of blood in comparison to 50–200 parasites/µL of blood of microscopy). Nevertheless, the authors mention that this technique requires some time to collect the data and perform the assays, and that the enhancement in the SERS peak is not dependent on hemozoin concentration. Yuen and Liu used Surface-enhanced Resonance Raman spectroscopy (SERRS) with Fe_3_O_4_@Ag nanoparticles (nanoparticles with an iron oxide core and silver shell), following magnetic field enrichment for hemozoin-based malaria detection [72]. The magnetic field concentrates the nanoparticles and the paramagnetic β-hematin at the laser spot, which increases the Raman signal [72]. The authors were able to detect 5 nM of β-hematin, the equivalent to 30 parasites/µL [72]. Nevertheless, the magnetic field could impact the variation of SERRS readings. Thus, later, the same group tested two methods: (1) silver nanoparticles mixed with *P. falciparum* and (2) silver nanoparticles produced inside the parasites, being in closer proximity with hemozoin [73]. The limit of detection of these methods were (1) 0.01% and (2) 0.00005%, which are equivalent to 100 and 5 parasites/µL of blood, respectively, quite competitive with optical microscopy and RDTs [73]. Although highly sensitive, these methods cannot provide direct quantification, due to errors including contamination with cell debris in the lysing process in method (1) and inconsistent distribution of hemozoin inside the parasite in method (2) [73]. However, in this paper, the authors report an easier nanoparticle preparation method that is low-cost and faster since they performed the SERS measurements on random locations instead of selected hot spots and with high sensitivity, which can be further improved by using paper-based microfluidics chip for sample preparation [73]. More recently, Yadav et al. [74] enhanced the SERS signal with silver nanorods (AgNRs) on 0.3 T neodymium magnetic substrates and an externally applied magnetic field. This ultra-highly sensitive technique allows the detection limit of the equivalent to less than 10 parasites/µL [74]. Another group reported a SERS biosensor (Figure 2B) tested with β-hematin, which exploits plasmon coupling features of gold nanoparticles to enhance the Raman signals, and their tunable SPR to the near-infrared region to facilitate biological analysis [75]. In this system, 20 µL of β-hematin is deposited on a gold film in close contact with gold nanoparticles embedded in transparent and flexible polydimethylsiloxane (PDMS). A 785 nm laser irradiates the system, originating SERS signals at 1623 cm^−1^, which are directly related to the amount of β-hematin deposited on the gold film [75]. Furthermore, the authors assured that hemoglobin cannot impact the response of SERS signals to β-hematin [75] and detected β-hematin concentrations of around 18.5 ± 4.5 and 51.5 ± 6.2 µM in healthy and sickle RBCs, respectively [75].

McBirney and colleagues designed, constructed and validated a portable, reagent-free magneto-optic technology for hemozoin detection [76,77]. This technology uses a 635 nm laser diode that emits in a 500 µL sample to a photodetector and a magnet [76]. The difference in the optical spectroscopy signal before and after applying the magnetic field indicates the level of infection [76]. This device was capable of detecting less than 8.1 ng/mL of β-hematin in 500 µL of whole rabbit blood, equivalent to less than 26 parasites/µL of blood (competitive with the 50–200 parasites/µL and 100–200 of microscopy and RDTs, respectively), and without any labelling [76,77]. Nevertheless, this system requires pre-treatment of the sample with ultrasound for blood lysis and the sample volume (500 µL) is not achievable with a finger prick [76]. The authors mention that further work will answer these limitations through the use of other techniques for blood lysis, reconfiguration of the sample cuvette and by using alternative photodetectors [76].

## 4. Lab-on-a-Chip and Other Microdevices for Hemozoin-Based Malaria Diagnosis

In addition to biosensors, other novel microdevices, in particular lab-on-a-chip devices, have been drawing attention due to their potential to be used at point-of-care malaria diagnosis [78]. These correspond to miniature portable devices that integrate several laboratory techniques, allowing the screening of different features to be performed together. Usually, these are coupled with microfluidic systems with reservoirs that allow the cells to be concentrated for a more specific and sensible detection.

Taylor and colleagues reported a simple to use, plastic hydrogel chip run on a portable real-time PCR [79]. This lab-on-a-chip is thermo-stable, is low-cost (USD 1 per test and less than USD 2000 for the real-time PCR compared with microscopy USD 0.12–0.40 per test and USD 700–3000 for the instrument for microscopy), uses a small sample volume (15 µL per test), provides the result in less than 2 h and is disposable [12,79]. This microdevice was tested in clinical samples and detected a limit of 2 parasites/µL of blood with high specificity (93.8%) and sensitivity (97.4%) compared with the conventional real-time PCR [79]. When testing an instrument with a LED excitation, the authors were able to increase specificity (100%) but not sensitivity (96.7%) [79]. The real-time PCR instrument requires the equivalent of a car battery as a power supply, which can be used in areas where the electricity supply is unstable [79]. The authors mention that a battery can be incorporated in the next generation of this lab-on-a-chip [79]. Furthermore, this micro-technology makes use of primers to amplify the 18S rRNA gene from *Plasmodium*, and by doing so, it was able to distinguish *P. falciparum* and *P. vivax* infections [79]. Despite not using hemozoin as a target, this microdevice sustains the applicability of lab-on-a-chip for malaria detection and it can be adapted for hemozoin targeting by using a different set of primers [79].

Recently, Hole and colleagues proposed an inductor on an FR-4 printed circuit board (PCB) and copper as a sensor for malaria screening [28]. The principle of work of this inductive sensor is based on effective relative permeability and on the inductance value of the core at the sensing coil, which is prone to changes when in the presence of paramagnetic hemozoin [28]. In fact, in the presence of hemozoin, inductance increases while resonance frequency decreases, allowing the detection of synthetic hemozoin in 12.7–25.4 pg, which is an amount equivalent to 25–50 parasites in 0.5 µL of phosphate-buffered saline (PBS), in a one-minute assay [28]. This value is competitive with the 50–200 parasites/µL and 100–200 parasites/µL of blood of microscopy and RDTs, respectively. Therefore, this sensitivity in such a small sample volume is promising for the early detection of the disease. Furthermore, the authors explain that they fabricated the inductor in a PCB, for this sensor to be low-cost, and that added a mask on top of the inductor for reusability and as a protective layer for the sensor, avoiding any damage to it [28]. Thus, this method is promising for malaria detection in the field, assuring an economical sensitive detection. Nevertheless, the use of copper limits the applicability of this sensor due to its easy and unavoidable oxidation and consequent loss of response over time.

**Figure 3:**
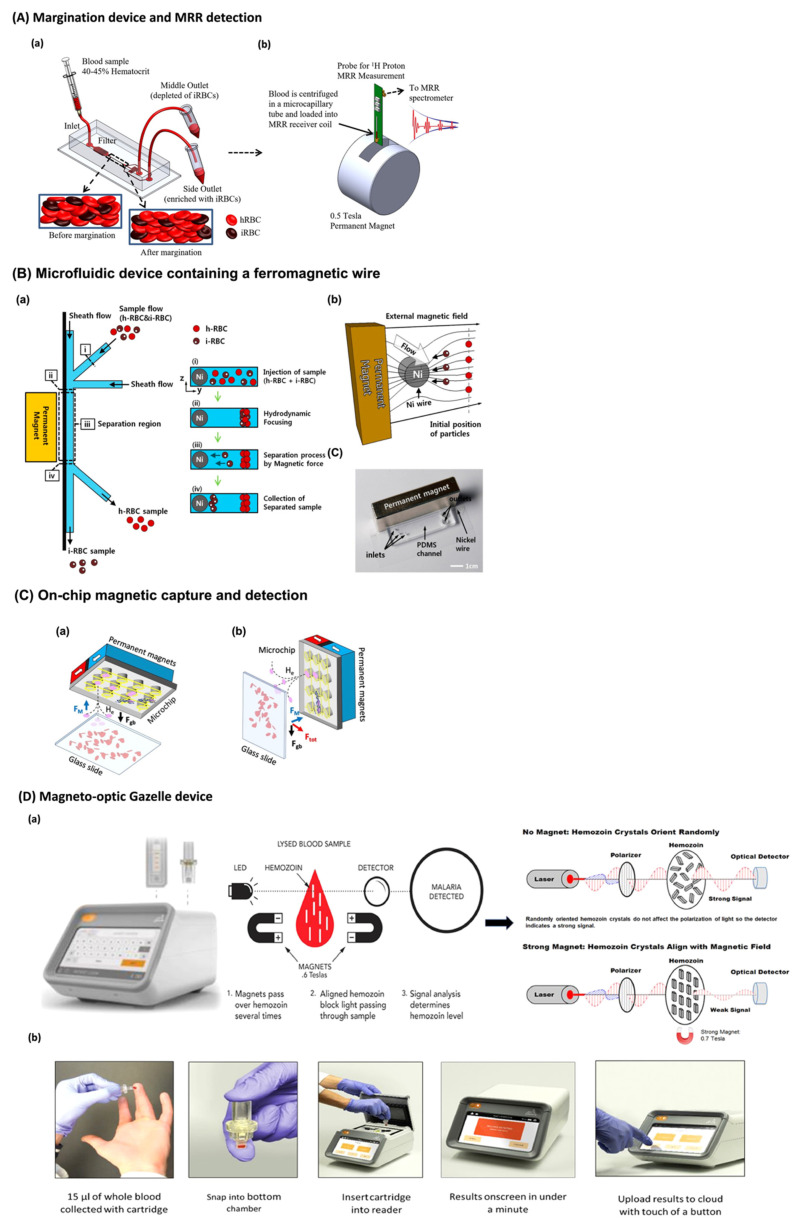
Example lab-on-a-chip and microdevices for malaria diagnosis based on hemozoin detection. (**A**) Schematic of the microfluidic margination device (a) and the benchtop MRR system (b); (**B**) Schematics of a microfluidic device containing a ferromagnetic wire fixed on a glass slide (a and b) and photograph of the system (c); (**C**) Horizontal (a) and vertical (b) configurations of an on-chip magnetic system; and (**D**) Photographic image of Gazelle and schematic of magneto-optic detection of hemozoin (a) and testing procedure (b). Reprinted with permission: (**A**) [42] copyright © 2015 The Authors, reproduced under a Creative Commons Attribution-NonCommercial-NoDerivatives (CC BY-NC-ND 4.0); (**B**) [90] copyright © 2013 American Chemical Society; (**C**) [91] copyright Licensee MDPI, Basel, Switzerland, under the Creative Commons Attribution License; and (**D**) [44] copyright © 2020 The Author(s). Published by Elsevier Ltd., under a Creative Commons Attribution-NonCommercial-NoDerivatives (CC BY-NC-ND 4.0). MRR: Magnetic Resonance Relaxation.

The relatively large paramagnetic susceptibility of hemozoin particles induces substantial changes in the transverse relaxation rate, *T2*, of proton nuclear magnetic resonance (NMR) of RBCs, which can be used to correlate with the presence of infected RBCs during malaria infection [80]. This idea was first pointed out by Karl et al. who show that it is possible to carry out NMR relaxometry on infected RBCs but concluded that it was unlikely to have enough sensitivity for malaria diagnosis in the field settings [81]. They demonstrate their studies using unprocessed raw blood [81]. Then, Peng and co-workers demonstrated that it was indeed possible to have a highly sensitive malaria diagnosis by focusing on the infected RBCs, using a simple trick of standard hematocrit centrifugation (from normal RBCs) [80,82,83,84,85]. The authors concluded this in their mouse studies where a highly sensitive detection compared to the current methods was reported (less than 10 parasites/µL versus 50–200 parasites/µL and 100–200 of microscopy and RDTs, respectively) [80]. Following this unprecedented development, several similar studies were reproduced [47,86,87,88], and new techniques were established to improve the infected RBCs separation (using microfluidics) and exploited for drugs studies [89]. In fact, Kong et al. [44] combined lab-on-a-chip microfluidics and magnetic resonance relaxometry (MRR) in order to accurately detect malaria infection (as shown in Figure 3A). The authors used margination-based microfluidics that separates infected and non-infected RBCs based on their different deformability. By doing so, infected RBCs were concentrated, facilitating infection detection. This was followed by infected RBCs lysis and MRR detection, based on paramagnetic hemozoin detection. By doing this, the authors were able to detect as low as to 0.0005% of parasitemia of early-stage *P. falciparum*-infected RBCs [44]. Based on the same RBCs count/µL of blood [71], the parasitemia is similar to the one of microscopy, 50 parasites/µL of blood. To avoid false-positive and -negative results, it is mentioned that each sample is analyzed 5–10 times in the MRR, which takes around 5–10 min [44]. The authors believe that both microfluidics design and MRR detection might be optimized to provide more sensitive and sensible results [44]. Furthermore, there is the possibility of miniaturizing both systems in a lab-on-a-chip, and despite the elevated cost of this (several thousands of USD), the cost per assay (less than USD 0.50) would be almost comparable with microscopy (USD 700–3000 for the instrument and USD 0.12–0.40 per test) and RDTs (USD 0.55–1.50 per test) [12,44].

The magnetic properties of hemozoin were also explored for a magnet-based microfluidic device. Nam et al. [90] developed a PDMS microchannel with three inlets and two outlets, fabricated by soft-lithography using SU-8, coupled to a nickel wire fixed on a glass slide (Figure 3B). In the presence of a permanent magnet, an external field of 0.6 T is created and causes the nickel wire to attract *P. falciparum* infected-RBCs, allowing their separation from the non-infected ones [90]. Thus, by using this microfluidic device, the authors were able to isolate and concentrate infected RBCs and suggest that the use of this before clinical diagnosis would increase its accuracy [90]. Nevertheless, the samples were separated with a recovery rate of approximately 73% and 98.3% corresponding to late- and early-stage parasites, respectively [90]. The authors mention that the efficiency of this device might improve by altering the distance between the nickel wire and the infected RBCs and by optimizing the microchannel outlet [90].

More recently, Milesi and co-workers developed a magnetophoretic on-chip system for malaria detection, also based on paramagnetic hemozoin detection (Figure 3C) [91]. The authors developed a silicon microchip with micro concentrators for the magnetophoretic capture of infected RBCs, and gold electrodes for measurement of the sample electrical impedance [91]. By doing so, the authors were able to selectively detect hemozoin crystals but noticed that the system could not easily distinguish malaria infection from met-hemoglobin, a hemoglobin paramagnetic state [91]. Quite recently, the same team optimized the silicon chip with nickel microcapillars that, in the presence of a magnetic field, should interact with the hemozoin crystals of infected samples [92]. This causes infected RBCs to become stacked. The authors used met-hemoglobin, converted from hemoglobin using NaNO_2_, to simulate infection [92]. In this model, with the proper agitation, 5 min are enough to attain 85% of capture efficiency [92]. Nevertheless, the same might not be achieved when using a real malaria sample. From a global perspective, electromagnetic sensing allows for highly sensitive and specific detection, as the use of a single excitation frequency (specific to the analyte) decreases the interferences from other molecules or media. However, the measurement of magnetic signals is also highly dependent on the temperature, so temperature compensation circuits must be taken into account [60].

Myrand-Lapierre and colleagues developed a multiplexed fluidic plunger to evaluate the deformability of RBCs through microscale funnels within a microchannel [93]. Later, the same team used this simple and inexpensive system to assess biophysical alterations in RBCs following hemin-induced oxidative stress [94]. One of the major sources of oxidative stress in the malaria parasite originates in the pathway of hemoglobin degradation to hemozoin formation as a result of iron oxidation [24,25]. Despite not measuring hemozoin directly, this system analysis an outcome of hemozoin formation and shows that hemin concentration correlates with RBCs deformability [94].

Recently Wang et al. [95] designed and fabricated in a PCB a surface acoustic wave (SAW) sensor, excited with a photo-acoustical signal. The team used a laser pulse into 2 µL of *P. falciparum*-infected RBCs and, in less than 2 min, were able to distinguish 1% of infected RBCs from non-infected RBCs [95]. The authors intend to integrate this sensor with a microfluidic system in order to increase the sensitivity through infected RBCs concentration [95]. Despite the low sample volume and rapid resolution time, considering an RBC count of 5 × 10^6^/µL of blood, the detection limit (100,000 parasites/µL of blood) is not competitive with microscopy and RDTs yet, nor do the authors specify whether this sensor operates based on hemozoin detection. Nevertheless, the sensitivity and specificity of SAW sensors might be improved by their coating with absorptive materials [96]. Despite being thermo-stable and not requiring high energy for operation, the durability of these sensors might be a challenge [96].

Furthermore, Graham et al. [97] proposed an ultrasensitive polymerization-based assay that allows hemozoin detection and quantification to be integrated into a microfluidic lab-on-a-chip device. The authors reported that solubilized hemozoin catalyzes the polymerization of N-isopropylacrylamide into poly N-isopropylacrylamide, resulting in liquid turbidity that can be optically quantified at 380 nm or 600 nm for up 4 h, as an indicator of malaria infection [97]. This polymerization process requires low-cost and thermo-stable reagents and allows the detection of 10 infected RBCs/µL of parasite-spiked full-blood on a small sample volume [97]. This value is quite competitive with the current diagnostic methods. Furthermore, the turbidity rate is proportional to the concentration of hemozoin, which makes the assay quantitative. More recently, the same group optimized the reaction conditions of the assay by using pyruvate, SDS and a 7.5 pH [98]. By doing so, the authors reduced the amplification time (the time for the reaction reach its maximum) from 37 ± 5 min to 3 ± 0.5 min, while keeping around the same detection limit and 95% confidence (1.06 ng/mL compared with 0.85 ng/mL, both equivalent to less than 10 infected RBCs/µL) [98]. It is interesting that the optimized conditions did not increase the sensitivity of the method but did significantly increase its performance time [98]. Despite requiring sample preparation for collection of blood and extraction of hemozoin, this improvement increases the applicability of the method.

Catarino and colleagues, after demonstrating that the absorbance spectra of synthetic hemozoin and hemoglobin is different, developed a first prototype of portable optical microdevice for hemozoin-based malaria detection and quantification [99,100]. The authors tested their system with 97 µL of whole blood samples mixed with a 1 µg/mL concentration of synthetic hemozoin in around 1 min analysis time [99]. Nevertheless, the need for a drop of blood is one disadvantage of this system and, thus, the authors have been exploring optical reflectance as an alternative non-invasive technique to be incorporated in a new microsystem [101,102,103].

Recently, Kumar et al. developed the magneto-optic Gazelle device (Figure 3D) for hemozoin detection [42,104]. Gazelle detects LED-emitted light into the sample in the presence and absence of a 55 T magnetic field [42]. The transmitted light is proportional to the amount of hemozoin in the sample and allows detection up to a limit of 50 parasites/µL of *P. falciparum* and 35 parasites/µL of *P. vivax* patients samples with 95% and 100% accuracy, respectively [42]. Gazelle is thermo-stable, battery operated, easy to use, low-cost (around USD 1 per test almost comparable with USD 0.12–0.40 of microscopy and USD 0.55–1.50 of RDTs) and fast (1 min in comparison with 30 min and 20 min for microscopy and RDTs, respectively) [42,104]. The device was tested on 262 patients in India and presented high sensitivity and specificity to diagnose the disease (98% and 97%, 82% and 99%, and 78% and 99% in comparison to microscopy, PCR and RDTs, respectively) [42]. Similar results were achieved in Brazilian Amazon and Peruvian Amazon Basian, which are *P. vivax*-predominant regions [105,106]. Nevertheless, Gazelle is not portable, and still requires a drop of blood for malaria detection (15 µL) and is not able to distinguish between species [42,104].

**Table 1:** Summarizes the main developments in biosensors, lab-on-a-chip devices and other microdevices for the detection of hemozoin and its variants.

Authors	Biosensor Type	Detection	Bio-Recognition Element	Analyte	Tested Sample	Limit of Detection	Detection Time	Ref.
Obisesan et al.	Electrochemical	3 electrode system, measured by cyclic voltammetry	Metal oxide nanoparticles of copper, iron and aluminum deposited on a gold electrode	β-hematin	Human non-malaria-infected urine samples, human malaria-infected serum, mice non-infected and infected serum, all mixed with β-hematin	*P. berghei* in infected mice’s serum samples: 3.60–4.8 mM (around 1.14 × 10^10^ parasites/μL of blood) *P. falciparum* in human blood serum samples: 0.65–1.35 mM (around 2.725 × 10^9^ parasites/μL of blood)	No information	[57]
Briand et al.	Optical	SPR-based sensor	Hemoglobin-polyacrylic acid	Heme	Heme solutions	2 µM *	Less than 10 min	[61]
Abshire et al.	Optical	FRET-based sensor	PfHRP-II	Heme	*P. falciparum*-infected RBCs	1.6 µM *	No information	[69]
Garret et al.	Optical	SERS	Gold-coated Graphium weiskei butterfly rings	Hemozoin	Lysed early-ring *P. falciparum*-infected RBCs	0.005% (equivalent to 50-500 parasites/µL of blood)	No information	[70]
Yuen and Liu	Optical	Magnetic enrichment followed by SERRS	Fe_3_O_4_@Ag nanoparticles	β-hematin	β-hematin resuspended in NaOH	5 nM (equivalent to 30 parasites/µL)	15 s exposure time	[72]
Chen et al.	Optical	SERS	Silver nanoparticles	Hemozoin	Silver nanoparticles mixed with *P. falciparum* and silver nanoparticles produced inside the parasites	0.01% and 0.00005% (equivalent to 100 and 5 parasites/µL of blood)	10 s exposure time	[73]
Yadav et al.	Optical	SERS and an externally applied magnetic field	Silver nanorods (AgNRs) on 0.3 T neodymium magnetic substrates	Hemozoin and Human deoxy-hemoglobin	Hemozoin and hemoglobin in PBS and deionized water; Fetal bovine seerum	equivalent to less than 10 parasites/µL	20–30 s integration time	[74]
Cai et al.	Optical	SERS biosensor	Gold nanoparticles embedded in PDMS	β-hematin	β-hematin and hemolyzed erythrocytes deposited on a gold film	18.5 ± 4.5 and 51.5 ± 6.2 µM in healthy and sickle RBCs	5 s for spectrum acquisiton time	[75]
McBirney et al.	Magneto-optic	635 nm laser diode that emits in the sample to a photodetector and a magnet	None	β-hematin	β-hematin in 500 µL of whole rabbit blood	8.1 ng/mL of equivalent to less than 26 parasites/µL of blood	No information	[76]
Taylor et al.		Lab-on-a-chip for DNA/RNA amplification	Master mix for amplification of the targeted DNA/RNA	18*S* rRNA gene	Frozen clinical samples of *P. falciparum*, *P. vivax* and *P. knowlesi*	2 parasites/µL of blood	Less than 2 h	[79]
Hole et al.	Inductive	Measurement of inductance/resonance frequency	None	Synthetic hemozoin	Synthetic hemozoin in PBS	12.7–25.4 pg in 0.5 µL of PBS (equivalent to 25–50 parasites/μL of blood)	No information	[28]
Peng et al.	Magnetic resonance	Magnetic resonance relaxometry detection	None	Hemozoin	early-stage *P. falciparum*-infected RBCs	Less than 10 parasites/µL in mouse studies culture	MRR detection: 5–10 min	[80,88]
Kong et al.	Magnetic	Lab-on-a-chip with MRR detection	None	Hemozoin	early-stage *P. falciparum*-infected RBCs	0.0005% of *P. falciparum* culture (equivalent to 50 parasites/µL of blood)	Separation process: 15 min MRR detection: 5–10 min	[44]
Nam et al.	Magnetic	Lab-on-a-chip and optical microscopy detection	None	Hemozoin	*P. falciparum*-infected RBCs	No information	No information	[90]
Milesi et al.	Magnetic/Electrical	Lab-on-a-chip with magnetophoretic capture and electrical impedance measurements	None	Hemozoin	Red blood cells treated and non-treated with NaNO_2_	No information	No information	[91]
Wang et al.	Photo-acoustic	Photo-acoust-excited surface acoust wave (SAW) sensor to be integrated with a microfluidic system	None	Not specified	*P. falciparum*-infected RBCs	1% of *P. falciparum*culture (equivalent to 100,000 parasites/µL of blood)	Less than 2 min	[95]
Graham et al.	Optical	Lab-on-a-chip with optical detection at 380 nm or 600 nm	N-isopropylacrylamide	Hemozoin	Hemozoin solutions in NaOH	10 infected RBCs/μL	37 ± 5 min	[97]
Raccio et al.	Optical	Lab-on-a-chip with optical detection at 380 nm or 600 nm	N-isopropylacrylamide	Hemozoin	Hemozoin solutions in NaOH	10 infected RBCs/μL	3 ± 0.5 min	[98]
Catarino et al.	Optical	Optical Absorbance	None	Synthetic hemozoin	Synthetic hemozoin diluted in whole blood	1 µg/mL	Around 1 min	[99]
Kumar et al.	Magneto-optic	Gazelle: LED-emitted light into the sample in the presence and absence of magnetic field	None	Hemozoin	*P. falciparum* and of *P. vivax* infected patient	50 parasites/μL and 35 parasites/μL of *P. falciparum and P. vivax* infected patients	Around 1 min	[42,105,106]
Lukianova-Hleb et al.	Photo-acoustic	Acoustic signal produced by laser induced vapor nanobubbles	None	Hemozoin	in vitro *P. falciparum*-infected RBCs and blood of *P. yoelii*-infected mice	0.0001% (in vitro); 0.00034% (in vivo); (equivalent to 10 parasites/µL and 17 parasites/µL for the in vitro and in vivo cultures)	No information	[46]

* As the authors measured heme concentration (an intervenient prior hemozoin formation), the values cannot be converted into parasite/µL.

## 5. Conclusions

Current malaria diagnostic methods are time-consuming and fail to detect low-level parasitemia in both asymptomatic and symptomatic patients. Nevertheless, diagnosis at the point-of-care is of vital concern since it provides information regarding parasitemia, allowing disease control, and might contribute to better patient care and treatment. Several methods have been targeting prompt and accurate malaria diagnosis, and despite quite promising approaches that have been developing, there is still room for improvement [11,12,13,14]. Globally, in the healthcare sector, biosensors have been offering ease-of-use at the point of care, sensitivity, specificity, high throughput and low fabrication costs, coupled to small sample volumes, rapid performance and low energy consumption [11,16,49,50]. These characteristics make biosensors a great alternative for combatting the emerging medical demands for point-of-care diagnosis, even in low-resource settings. Regarding malaria, several biomarkers have been targeted for biosensors detection [11,16,17].

Particularly, the heme detoxification pathway, which goes from hemoglobin degradation to hemozoin formation, presents several players that could be exploited for biosensors-mediated malaria diagnosis. Hemozoin is formed as parasites invade the host RBCs, its concentration correlates with disease progression, and it is present in the malaria transmitters gametocytes. Therefore, it is a unique biomarker for infection identification. Moreover, the hemozoin crystal itself presents specific features that, coupled with the technology of biosensors, could result in the development of an indispensable device that would fulfil all the medical demands for malaria management and elimination. Consequently, in recent years, hemozoin has been actively explored for diagnosis [12,39,40,46,103,107]. However, to the best of our knowledge, hemozoin was not greatly explored for biosensors detection yet, most likely due to the challenge of finding proper bio-recognition elements with high specificity for its detection, as well as proper sensing mechanisms. Additionally, the heme detoxification pathway that culminates in hemozoin formation has not been completely resolved yet. Additionally, in fact, the players in hemozoin formation have not been identified so far. This, in parallel with the current challenges of the different biosensing techniques (discussed in this manuscript), justifies the lack of bio-recognition elements for integration in biosensors for the detection of hemozoin.

Therefore, the current biosensors based on this biomarker still hinder the success of biosensors for the aimed simple, sensitive, and low-cost malaria detection. Nevertheless, an instrument might be expensive at first (when buying) but low-cost over time, bringing the potential for point-of-care diagnosis. For example, Kong et al. report that their device might cost several thousand USD, but then the cost per assay would be less than USD 0.50 [44]. This is quite comparable with microscopy, in which USD 700–3000 are required for the instrument and USD 0.12–0.40 per test. In this specific case, the authors would require a capillary to collect and analyze the sample, not requiring additional disposal items (such as the glass slide and Giemsa-staining required by microscopy). The same might apply to the other mentioned techniques. Furthermore, since hemozoin is not present in other fluids (other than blood), it requires a finger prick for blood collection, which is one of the biggest disadvantages of current diagnostic methods. The need for a finger prick for blood collection results in bio-hazard items that are hard to handle in endemic areas and that limit diagnosis based on hemozoin detection since only early-stage parasites carrying lower amounts of hemozoin circulate in peripheric blood [108]. Lab-on-a-chip devices, in addition to contributing to easy portability and lower costs, are useful in this situation. Microfluidic systems with reservoirs allow infected and non-infected RBCs to separate and concentrate, most likely increasing the sensitivity and specificity of the detection. These systems are, therefore, useful prior to malaria detection with other technology. Furthermore, the specificity of microfluidic systems for infected and non-infected RBCs separation might improve when coupled, for instance, with a magnet. In the presence of a magnetic field, the magnetic hemozoin crystallites interact with the field being attracted, whereas non-infected RBCs flow through. This technology also enables the processing of samples with low volumes, high-throughput, and low processing times. Moreover, the possibility to incorporate/adapt a battery in a lab-on-a-chip device is of interest, particularly in areas where the power supply is unstable or lacks access to proper infrastructures.

The currently proposed biosensors and lab-on-a-chip devices for hemozoin detection show interesting results (e.g., low detection limit or speed), despite requiring significant performance improvement for field applicability. Thus, improving the current sensing strategies and unveiling the hemozoin formation pathway might render targets for the development of both antimalarial drugs and new biosensors and lab-on-a-chip devices for diagnosis. Therefore, by considering the currently available literature, we believe that biosensors and lab-on-a-chip devices based on hemozoin detection might be, indeed, the future in malaria control and elimination.
